# Parasitic pneumonia in roe deer (*Capreolus capreolus*) in Cornwall, Great Britain, caused by *Varestrongylus capreoli* (Protostrongylidae)

**DOI:** 10.1186/s12917-018-1525-x

**Published:** 2018-06-22

**Authors:** Victor R. Simpson, Damer P. Blake

**Affiliations:** 1 Wildlife Veterinary Investigation Centre, Chacewater, Truro, Cornwall, TR4 8PB UK; 2 Pathobiology and Population Sciences, Royal Veterinary College, Hawkshead Lane, North Mymms, Hertfordshire, AL9 7TA UK

**Keywords:** Roe deer, *Capreolus*, *Varestrongylus*, Pneumonia, Protostrongylid

## Abstract

**Background:**

Roe deer (*Capreolus capreolus*) became extinct over large areas of Britain during the post mediaeval period but following re-introductions from Europe during the 1800s and early 1900s the population started to recover and in recent decades there has been a spectacular increase. Many roe deer are shot in Britain each year but despite this there is little published information on the diseases and causes of mortality of roe deer in Great Britain.

**Case presentation:**

The lungs of two hunter-shot roe deer in Cornwall showed multiple, raised, nodular lesions associated with numerous protostrongylid-type nematode eggs and first stage larvae. There was a pronounced inflammatory cell response (mostly macrophages, eosinophils and multinucleate giant cells) and smooth muscle hypertrophy of the smaller bronchioles. The morphology of the larvae was consistent with that of a *Varestrongylus* species and sequencing of an internal transcribed spacer-2 fragment confirmed 100% identity with a published Norwegian *Varestrongylus* cf. *capreoli* sequence. To the best of the authors’ knowledge this is the first confirmed record of *V. capreoli* in Great Britain. Co-infection with an adult protostrongylid, identified by DNA sequencing as *Varestrongylus sagittatus*, was also demonstrated in one case.

**Conclusions:**

Parasitic pneumonia is regarded as a common cause of mortality in roe deer and is typically attributed to infection with *Dictyocaulus* sp. This study has shown that *Varestrongylus capreoli* also has the capability to cause significant lung pathology in roe deer and heavy infection could be of clinical significance.

## Background

Roe deer (*Capreolus capreolus*) and European red deer (*Cervus elaphus elaphus*) are the only species of deer native to the British Isles. Although historically widespread, roe deer became extinct over large areas of Britain during the post mediaeval period, particularly in Wales and the English Midlands [1, 2]. However, following re-introductions from France, Germany, Austria and Siberia during the 1800s and early 1900s [1, 3] roe deer populations in Britain started to recover and it is now believed that all roe deer in southern England are derived from animals introduced from Europe [2]. In recent decades there has been a spectacular increase in the British roe deer population [4] and, with the rate of range expansion recently estimated at 2.3%, per annum, this is expected to expand further for the foreseeable future [5]. The factors that are driving this and other deer population trends in Britain are poorly understood and there is a need for an evidence-based understanding of the mechanisms involved [6]. Increased roe deer density has previously been implicated in the enhanced spread of pathogens such as *Mycobacterium bovis* [7] and might increase the risk of chronic wasting disease (CWD) transmission should it reach the UK [8]. When combined with climate changes which may support larger mollusc populations [9], and a concomitant enhanced risk of transmission of mollusc-vectored pathogens, there is a greater risk that parasites such as *Varestrongylus capreoli* will become established in the UK. Many roe deer are shot in Britain each year for human consumption and also to limit the damage they do to commercial woodland [10]. Despite this, there is little published information on the diseases and causes of mortality of roe deer in this country.

## Case presentation

### Samples and sample preparation

The lungs and, in one case the majority of the lung lobes, from two adult male roe deer were submitted to the Wildlife Veterinary Investigation Centre by hunters in April 2015 and April 2017 following the observation of gross abnormalities for the purpose of food safety. Both deer were shot by licenced marksmen as part of estate management strategies at locations denoted by National Grid Reference numbers SX08 64 and SW84 45, respectively. One animal (case #1) had been shot because it was lame, having suffered a recent amputation of the left forelimb distal to the carpus; despite this injury it was in quite good physical condition. The second animal (case #2) was shot for human consumption and was judged to be in good body condition. The lungs in each case were subjected to gross examination and showed multiple swellings in the parenchyma. In order to check for the possible presence of parasite eggs or larvae impression smears from the cut surface of the swellings were made on to microscope slides; a small amount of normal saline was added, a coverslip applied and the specimen examined by direct light microscopy. Duplicate impression smears were mounted in dilute Lactophenol Cotton Blue to clear and stain the characteristic features of any parasitic forms present. In case #1 only an additional smear was air-dried, heat-fixed and stained by Ziehl-Neelson in order to check for the presence of acid-fast organisms such as *Mycobacterium* sp.. Larvae and parasite appendages were measured using an eye-piece micrometer calibrated against a stage micrometer. Representative sections through several parenchymal swellings of both sets of lungs were placed in 10% neutral buffered formalin, dehydrated through graded alcohols, embedded in paraffin wax, sectioned at 5 μm and stained by haematoxylin and eosin (H&E) and periodic acid-Schiff (PAS). Additionally, lungs from case #2 were examined by fine blunt dissection for the presence of adult nematodes. Fragments of dissected lung were pooled, placed in a glass beaker with 2 L of tap water, thoroughly agitated, left to settle and then the sediment examined for parasites or fragments.

### Gross pathology

**Fig. 1:**
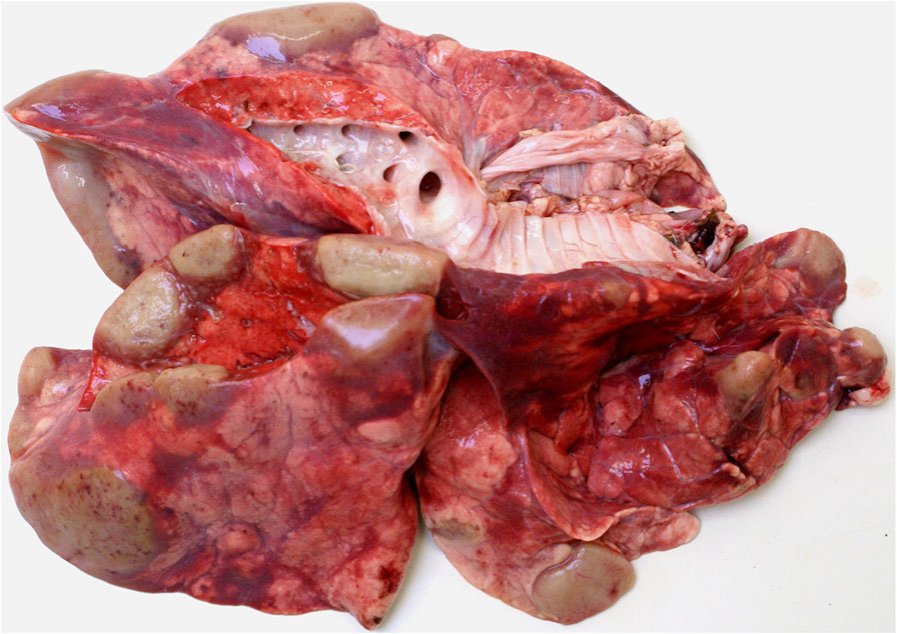
The gross appearance of the lungs in case #1 showing multiple pale tan nodular swellings in the pulmonary parenchyma

The lungs in case #1 showed multiple tan-coloured, nodular, roughly 2–5 cm diameter swellings located mostly along the margins of the lobes. They were clearly demarcated from the lung parenchyma, had a firm, uniform consistency and frequent small ecchymotic haemorrhages. Also present in the parenchyma were localised areas of atelectasis and haemorrhage (Fig. 1). There was a small number of adult *Dictyocaulus* sp. nematodes present in the bronchi and larger bronchioles. The nodular lung lesions in case #2 were very similar to those in the first case but were often surrounded by a zone of parenchyma that was much paler than elsewhere in the lung.

### Bacteriology

No acid-fast organisms were seen in a Ziehl-Neelsen stained, air-dried and heat-fixed impression smear from the cut surface of a lung nodule in case #1.

### Histopathology

**Fig. 2:**
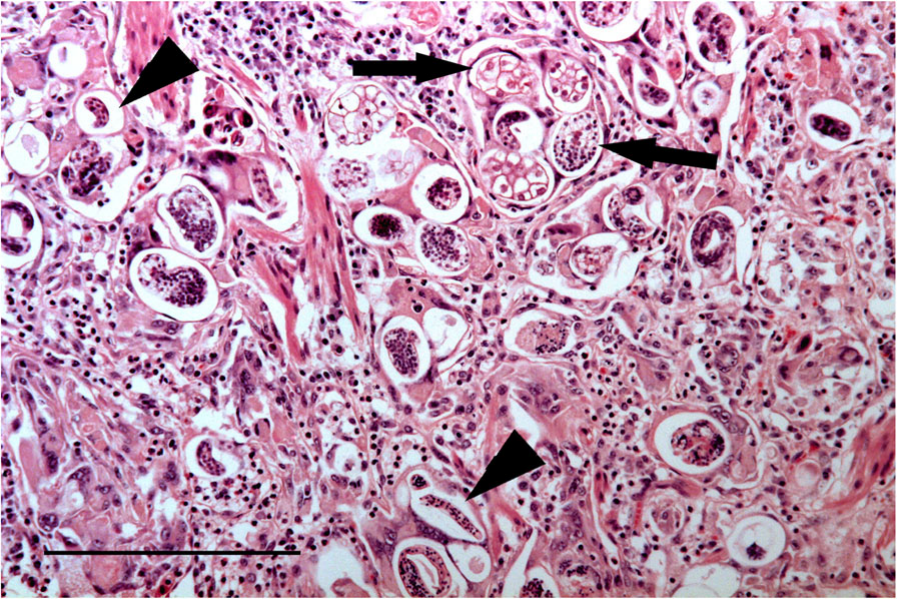
Histological section through a nodular swelling in the lungs of case #1. Note the eggs in various stages of development (arrows) and first stage larvae (arrow heads) H&E stain. Bar =200 μm

**Fig. 3:**
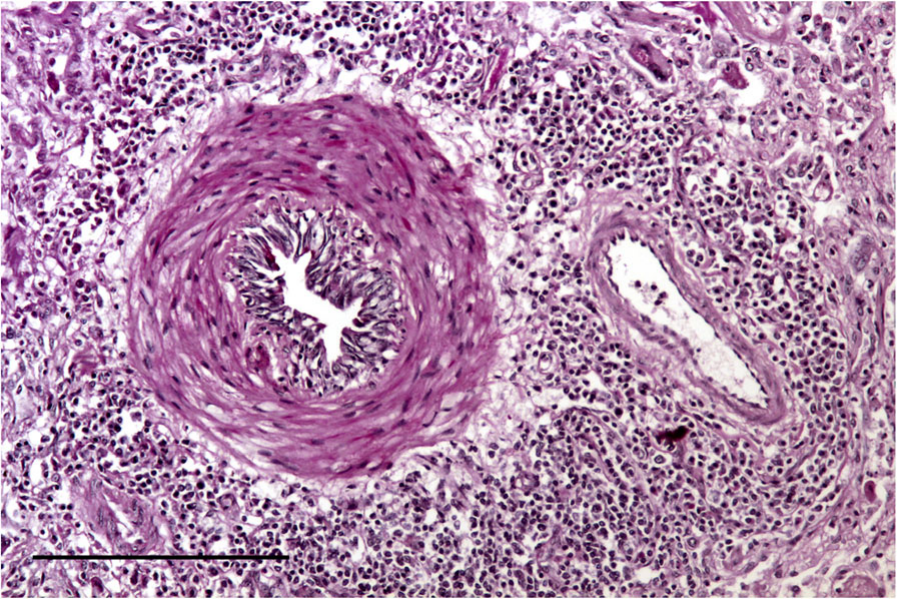
Histological section through the lung of case #1 showing severe smooth muscle hypertrophy of a small bronchiole and a surrounding rim of lymphocytes. PAS stain. Bar =200 μm

The nodular lesions in both cases showed dense consolidation with very large numbers of nematode eggs present in various stages of development together with numerous larvae in the bronchioles and the alveolar lumena. Associated with this was a large number of inflammatory cells, mostly macrophages, eosinophils and multinucleate giant cells, and fibrosis of alveolar walls (Fig. 2). The smaller bronchioles showed pronounced smooth muscle hypertrophy and were surrounded by a thick layer of lymphocytes (Fig. 3). No adult nematodes were seen in the nodules or in unaffected lung parenchyma.

### Parasitology

There were moderate numbers of adult *Dictyocaulus* sp. in the bronchi and bronchioles of case #1 but none were seen in case #2.

**Fig. 4:**
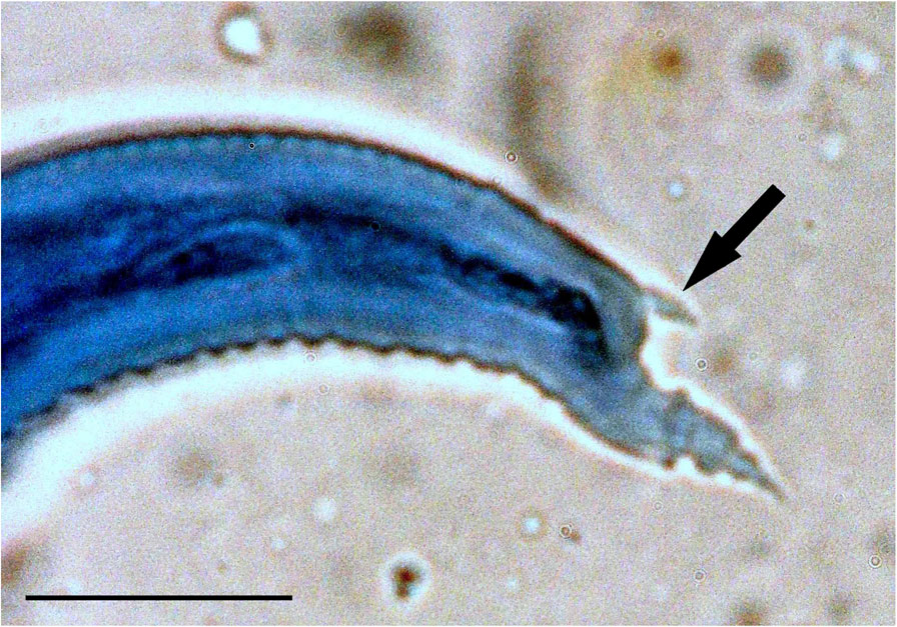
High power view of the tail appendage with cuticular ridges and the dorsal spine (arrow) of a first stage larva in an impression smear from a lung nodule in case #2. Lactophenol Cotton Blue stain. Bar =25 μm

In both cases microscopic examination of wet impression smears from the cut surface of the nodules revealed numerous first stage nematode larvae and embryonated eggs. The larvae had a dorsal spike at the base of the tail appendage and the appendage itself had well-developed cuticular folds (Fig. 4). The larvae in case #2 were measured and the mean dimensions (*n* = 10) were length 294.4 μm (standard error 3.3) and width 18.8 μm (0.7).

**Fig. 5:**
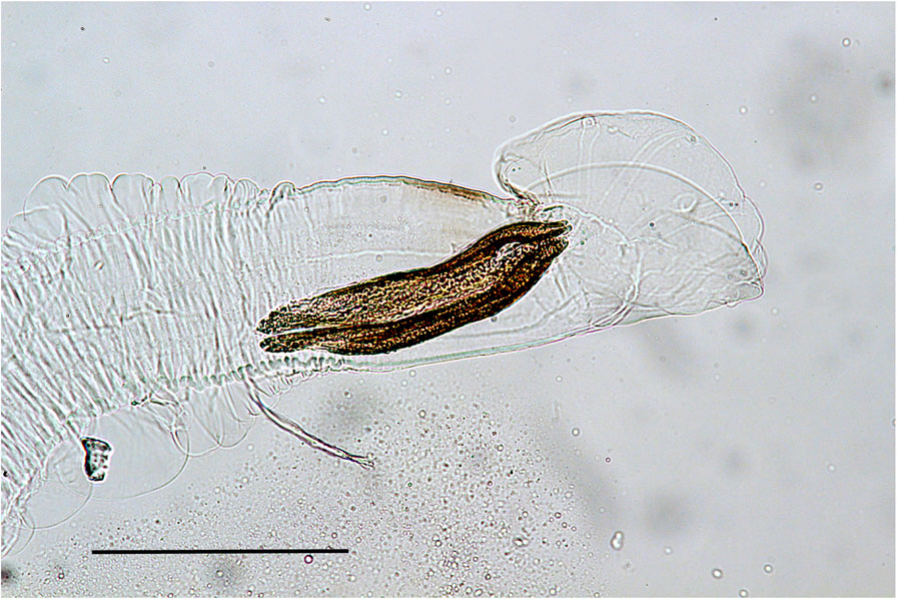
Caudal end of the male protostrongylid recovered following blunt dissection of lung in case #2. The spicules are tubular, equal size and symmetrical and the bursal rays do not extend fully to the margins. Unstained. Bar =200 μm

Blunt dissection of lung parenchyma was carried out in case #2 only. Examination of the sediment after washing the pooled fragments of lung tissue revealed a single male nematode measuring approximately 5 mm in length. It had well developed bursae with radiating rays that did not extend fully to the margins. The spicules were tubular, equal, symmetrical and yellowish brown and measured 258.5 μm (Fig. 5).

### Molecular biology

Total genomic DNA was extracted from (i) pooled L1 larvae recovered from an additional unfixed impression smear sample from case #2 only and (ii) a single adult male preserved in 70% (*v*/v) ethanol, using a Qiagen DNeasy Blood and Tissue kit following the animal tissues spin column protocol as described by the manufacturer (Qiagen, Hilden, Germany). A fragment of the internal transcribed spacer (ITS)-2 sequence within the nuclear ribosomal DNA was amplified in duplicate and sequenced from each sample using the generic pan-nematode primers NC1 (5′-ACGTCTGGTTCAGGGTTGTT-3′) and NC2 (5′-TTAGTTTCTTTTCCTCCGCT-3′) [11] to confirm parasite identity. Briefly, each PCR reaction contained 3 μl template DNA, 1.5 μl of each of the relevant forward and reverse primers (10 μM stock; Sigma-Aldrich, Poole, UK) and 20.0 μl of MyTaq × 2 mastermix (Bioline, London, UK), made up to a final volume of 40 μl with molecular grade water (Sigma). Molecular grade water was used as a no template negative control. Thermal cycler parameters were: 1 x initial denaturation at 95 °C for 2 min, followed by 35 x (denaturation 30 s at 95 °C, annealing 30 s at 52 °C, extension 30 s at 72 °C), followed by a final extension phase of 72 °C for 7 min. PCR amplicons were resolved by agarose gel electrophoresis using a 1% (*w*/*v*) UltraPure agarose gel in 1× Tris-borate-EDTA buffer (TBE; all Sigma), including 0.01% (*v*/v) SafeView nucleic acid stain (NBS Biologicals). The results of electrophoresis were visualised using a U:Genius Gel Documentation System(Syngene). PCR amplicons were purified using a PCR purification kit (Qiagen) and sequenced on both strands using the same primers employed in their original amplification (GATC Biotech, Konstanz, Germany). Sequence assembly, annotation, and interrogation were undertaken with CLC Main Workbench v6.0.2 (CLC Bio, Aarhus, Denmark) using BLASTn against the GenBank non-redundant database to assign identity.

Following these methods partial ITS-2 sequences were derived from L1 larvae and the single adult male worm. In total 449 bp high quality sequence was produced in duplicate from the L1 larvae, with 100% sequence similarity between duplicates (available under accession number LT962658). Comparison with the GenBank non-redundant nucleotide database identified the highest sequence similarity with *V.* cf. *capreoli* (accession number KJ452176.1; 100% coverage, 100% identity). Separately, 480 bp sequence was produced in duplicate from the adult male worm (LT962657), with greatest similarity to *Varestrongylus sagittatus* (KJ439592.1; 100% coverage, 100% identity). Comparison with published *V. sagittatus* ITS-2 sequences KJ439592–4 and KJ439596–7, revealed 15 copies of the degenerate TCG/CCG triplet repeat, which may provide future value as a genetic marker.

## Discussion

The protostrongylid nematode *Varestrongylus capreoli* is a recognised cause of lung lesions in roe deer in Europe [12–15]. However, prior to the present study it does not appear to have been recorded in roe deer in the British Isles. A histopathological study of roe deer lungs in Scotland described infection with an unidentified protostrongylid nematode but as examination was confined to histopathology no description of the parasite other than the appearance of the eggs and larvae was provided [16]. The paper does not include a description of the gross pathology of the lungs so it is not apparent whether or not there were nodular lesions similar to those seen in the present study. However, the histopathological findings closely resembled those seen in the present Cornish cases with larvae and eggs filling alveolar lumena accompanied by eosinophils, giant cells, alveolar macrophages and some neutrophils, supporting association of the inflammatory response observed with the parasite infection. As in the present study, severe muscular hypertrophy of the smaller bronchiolar walls was a prominent feature [16].

A notable feature in both studies is the absence of adult nematodes in the histological sections. However, adults have been recorded in both alveoli and bronchioles of roe deer elsewhere in Europe [14]. A possible explanation for their absence in some instances is that they may survive for only a short while after egg laying and are phagocytosed. A similar situation exists with *Capillaria hepatica* infection where eggs often remain in the hepatic parenchyma after the adults have died and been resorbed (Simpson, unpublished data). However, adults of *V. sagittatus* remain viable for several years [17] and it seems unlikely that *V. capreoli* would be markedly different from this closely related species. Both deer in the present study were killed in April and showed evidence of active infection with eggs and larvae in varying stages of development. The seasonality of infection for *V capreoli* does not appear to have been documented but *V. sagittatus* infection of maral deer (*Cervus elaphus maral*) peaks during winter and spring [17]; this could possibly be in relation to a period when either the molluscs which act as intermediate hosts become most active or when ingestion of infective larvae is most common. It has been postulated that parasites could infect deer during feeding on dry grass and short shrubs covered by snow in which hibernating snails occur [12, 14]. Also, the presence of immature forms in the lungs could represent a latent phase of infection [12, 14].

The gross appearance of the nodular lung lesions in the present study bore a superficial resemblance to lung lesions due to tuberculosis in roe deer in Spain and Italy [7]. The lesions in those cases included foci in the pulmonary parenchyma that were occasionally calcified and surrounded by a thin layer of epithelioid and multinucleated giant cells. Although no such lesions were seen in the roe deer infected with *Varestrongylus sp.* it does illustrate the need to examine lung lesions in roe deer for evidence of infection by mycobacteria.

Many conservationists may welcome the re-establishment of a former native species after it had become extinct or extremely rare. However, the recent recolonization of England by roe deer has been dramatic and shows no sign of reducing. This raises concern about an increased risk of disease transmission, such as tuberculosis, due to animals living at a higher population density [7]. In addition there is growing concern that climate change is likely to influence the geographic distribution of diseases such as bluetongue and Lyme disease [6]. There is a need to be aware of these factors from both a conservation viewpoint and also from a zoonotic perspective.

Identification of protostrongylid larvae by morphological features is not generally possible. The first stage larvae of species of the subfamilies Muelleriinae, Elaphostrongylinae and Varestrongylinae all have a dorsal spine and they also have a similar tail appendix [18]. However, the larvae of the various species have a recognised size range and this can assist in identification. In the present study the mean length of the larvae was 294.4 μm and this was within the recorded range of 285–341 μm for *Varestrongylus capreoli* [17], although larvae for other *Varestrongylus* spp. fall within the same or similar ranges [15]. Identification was confirmed by sequencing of the ITS-2, which gave a 100% match to a published Norwegian *Varestrongylus* cf. *capreoli* sequence.

The adult male protostrongylid recovered after blunt dissection of the lung was identified by DNA sequencing as *Varestrongylus sagittatus*. This is known to be a common lungworm of European red, maral and fallow (*Dama dama*) deer in Europe and Asia where it produces small nodules in the lungs, similar to those caused by *Muellerius capillaris* in sheep and goats [19]. *Varestrongylus sagittatus*, described using the synonym *Bicaulus sagittatus*, has also been recorded in Britain where it was found in a roe deer buck shot in the New Forest [20]. No morphological or other data was provided to confirm the identification. *Varestrongylus sagittatus* is one of a group of seven *Varestrongylus* species which are characterised by having spicules greater than 200 μm. However, although they measured 258.5 μm in the roe deer specimen in this study, this is significantly shorter than the range normally quoted for *V. sagittatus* (325–433.8 μm) [15]. The total length of the nematode at around 5 mm is also much less than the accepted range for an adult male *V. sagittatus* (14.5–33.8 mm, as well as for a number of other species such as *V. alpenae* (13–15 mm), *V alces* (11.36–14.7 mm) and *V capricola* (15–18 mm) [15]. A possible explanation for the small size of *V. sagittatus* in this study is that this is typically a parasite of red or fallow deer, not roe deer, and its development may have been restricted in an atypical host.

## Conclusions

Verminous pneumonia due to infection with *Dictyocaulus* sp. is considered to be an important cause of mortality in roe deer in Britain [2, 21] and in some areas has been considered to act as the main factor controlling populations [21]. This study has shown that *Varestrongylus capreoli* also has the capability to cause significant lung pathology in roe deer. There is clearly a need to consider the possible involvement of this parasite when investigating lung disease in roe deer, especially as mixed infections with the more obvious *Dictyocaulus* parasite may occur resulting in under diagnosis of *V. capreoli*.
